# Stem cells isolated from adipose tissue of obese patients show changes in their transcriptomic profile that indicate loss in stemcellness and increased commitment to an adipocyte-like phenotype

**DOI:** 10.1186/1471-2164-14-625

**Published:** 2013-09-16

**Authors:** Blanca Oñate, Gemma Vilahur, Sandra Camino-López, Alberto Díez-Caballero, Carlos Ballesta-López, Juan Ybarra, Fabrizio Moscatiello, Javier Herrero, Lina Badimon

**Affiliations:** 1 Cardiovascular Research Center, CSIC-ICCC, Hospital de la Santa Creu i Sant Pau, IIB-Sant Pau, Barcelona, Spain; 2 Quirúrgica cirujanos Asociados, Centro Médico Teknon, Barcelona, Spain; 3 C.L.B Centro laparoscópico Doctor Ballesta, Barcelona, Spain; 4 Resistencia a la Insulina SL, Barcelona, Spain; 5 Department of Plastic Surgery, Centro Médico Teknon, Barcelona, Spain

**Keywords:** Human adipose-derived stem cells, Subcutaneous adipose tissue, Cardiovascular risk factors, Transcriptome, Inflammatory genes

## Abstract

**Background:**

The adipose tissue is an endocrine regulator and a risk factor for atherosclerosis and cardiovascular disease when by excessive accumulation induces obesity. Although the adipose tissue is also a reservoir for stem cells (ASC) their function and “stemcellness” has been questioned. Our aim was to investigate the mechanisms by which obesity affects subcutaneous white adipose tissue (WAT) stem cells.

**Results:**

Transcriptomics, in silico analysis, real-time polymerase chain reaction (PCR) and western blots were performed on isolated stem cells from subcutaneous abdominal WAT of morbidly obese patients (ASCmo) and of non-obese individuals (ASCn). ASCmo and ASCn gene expression clustered separately from each other. ASCmo showed downregulation of “stemness” genes and upregulation of adipogenic and inflammatory genes with respect to ASCn. Moreover, the application of bioinformatics and Ingenuity Pathway Analysis (IPA) showed that the transcription factor Smad3 was tentatively affected in obese ASCmo. Validation of this target confirmed a significantly reduced Smad3 nuclear translocation in the isolated ASCmo.

**Conclusions:**

The transcriptomic profile of the stem cells reservoir in obese subcutaneous WAT is highly modified with significant changes in genes regulating stemcellness, lineage commitment and inflammation. In addition to body mass index, cardiovascular risk factor clustering further affect the ASC transcriptomic profile inducing loss of multipotency and, hence, capacity for tissue repair. In summary, the stem cells in the subcutaneous WAT niche of obese patients are already committed to adipocyte differentiation and show an upregulated inflammatory gene expression associated to their loss of stemcellness.

## Background

White adipose tissue (WAT), traditionally viewed as an energy storage tissue, is now recognized as an endocrine organ that harvests and serves as repository of mesenchymal stem cells, the adipose-derived stem cells (ASC), for physiological cell renewal and spontaneous repair. Henceforth, these ASC have the potential to differentiate towards multiple tissue lineages, produce a large variety of growth factors and present immunomodulatory properties [1]. Mesenchymal stem cells (MSC) homing from bone marrow to peripheral tissue is probably the most specialized organ repairing process. During the last few years it has been possible to isolate MSC from different tissues [2].

Obesity, a chronic pathological condition, is a risk factor for cardiovascular disease, but paradoxically, it seems to protect against morbi/mortality from heart failure [3]. The hypothesis of a repository of stem cells in WAT of heart failure-obese patients that may serve as a source for spontaneous repair has never been tested. It has been recently suggested that any alteration of the stem cell homeostasis by constant and repetitive trauma, and chronic disease could provoke a persistent disequilibrium inside the stem cell reservoir leading to an irreversible and premature decrease of the stem cells regenerative potential reducing their “stemcellness” [2]. Because a growing body of evidence suggests that depot-specific variations in resident stem cells are retained despite in vitro culture processes [3], we investigated the isolated stem cells from the adipose tissue niches to identify their changes due to obesity. Indeed, we have previously reported that adipose-derived stem cells (ASC) from obese patients show a differentiation potential and are less proangiogenic than ASC from non-obese individuals [4]. However, how obesity affects ASC and which are the affected genes remain unclear.

Differences in gene expression between subcutaneous and visceral WAT have been reported. As such, genes involved in energy homeostasis and adipogenesis are profoundly altered in obese WAT [5-8]. Additionally, in obese individuals’ mature adipocytes, the major WAT-cell types have shown to present an altered inflammation-related gene expression profile [9]. In addition, studies in subcutaneous preadipocytes from type 2 diabetes patients and obese Pima Indian subjects have reported diminished expression of genes involved in differentiation and an upregulation of inflammation-related genes [10,11]. Moreover, obesity has shown to dysregulate the stemness gene network of omental-ASC [12]. However, there are few studies investigating how obesity affects the transcriptome of resident stem cells reservoir.

In this study, we investigated the gene expression profile and the involved biological functions in ASC of obese patients using a systems biology approach. ASC were isolated from WAT to avoid the contribution of differentiated cells in subcutaneous WAT to the transcriptomic signature. We studied gene expression profile of isolated subcutaneous ASC from patients with obesity and clustering of cardiovascular risk factors and ASC from non-obese healthy individuals. We used a bioinformatic approach with “in silico” analysis to identify biological functions and target genes potentially altered in ASC. Further validation of candidate genes has identified potential targets associated to a reduced regenerative potential.

## Results

### Patient demographics and ASC phenotypic characterization

ASC were harvested from morbidly obese patients [Body mass index (BMI) = 44.44 ± 1.29 kg/m^2^], and non-obese individuals (BMI = 22.26 ± 0.88 kg/m^2^). Although both groups were comparable in age (morbidly obese patients = 41.56 ± 3.07 years; non-obese individuals = 38.28 ± 2.48 years), morbidly obese patients showed significantly (p < 0.05) elevated glucose (142.13 ± 11.95 mg/dl vs 61.54 ± 7.35 mg/dl), creatinine levels (0.83 ± 0.07 mg/dl vs 0.55 ± 0.1 mg/dl) and total cholesterol/high density lipoprotein cholesterol (HDL) ratio (5.92 ± 0.48 vs 3.98 ± 0.38), and lower HDL cholesterol levels (33.2 ± 3.05 mg/dl vs 48.32 ± 2.82 mg/dl) as compared to non-obese individuals. No differences were detected in triglyceride, total cholesterol, low density lipoprotein cholesterol (LDL), urea, total proteins and hepatic enzymes levels.

Flow cytometry analysis of isolated ASC from morbidly obese patients (ASCmo) and non-obese individuals (ASCn) revealed similar ASC phenotype in both groups of cells at passage 3, when the transcriptomic analysis was performed. Both cell types strongly expressed typical ASC surface antigens such as CD90 (Thy-1 cell surface antigen), CD29 (Integrin beta 1), CD44 (Hyaluronic acid receptor), and CD105 (Endoglin) and were negative for CD45, a hematopoietic cell marker, and for CD34, a marker of bone marrow cells, hematopoietic stem cells, endothelial progenitor cells and muscle stem cells (Additional file 1: Table S1). Interestingly the flow cytometry analysis at passage 0 demonstrated that passage 3 cells had similar ASC surface markers levels (CD105, CD29, CD90 and CD44) with respect to passage 0 cells. However, a significant reduction in CD34 and CD45 was found indicating that the passage 3 cells used for transcriptomic analysis were a homogeneous ASC population (Additional file 1: Table S1).

### Transcriptomic analysis

In order to compare gene expression patterns between ASCmo and ASCn, a comprehensive transcriptomic analysis was performed using Affymetrix whole-transcript expression array. To visualize gene expression data we performed Principal Component Analysis (PCA) that demonstrated the independence of transcriptomic signature in ASCmo and ASCn (Additional file 2: Figure S1). To further explore genes differentially expressed between ASCmo and ASCn, we used modified analysis of variance (ANOVA) analysis. With a p-value < 0.2, a 60% False Discovery Rate (FDR) and a fold change > 1.5, 637 differentially expressed transcripts were detected while with a p-value < 0.1, a 60% FDR and a fold change > 1.5, 457 gene transcripts were detected (Additional file 3: Figure S2). Among the 637 detected genes, 319 exhibited an increased expression in ASCn (fold change range: 1.5 to 7.2) while 318 were overexpressed in ASCmo (fold change range: 1.5 to 18).

### ASCmo and ASCn gene expression patterns

**Figure 1:**
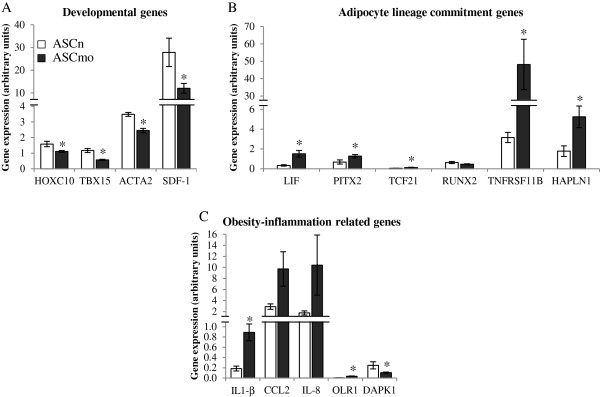
**Validation of genes of the microarray analysis by real time PCR.** Genes are grouped by **(A)** involved in developmental processes, **(B)** adipocyte commitment, and **(C)** in the obesity-inflammation axis. (* p < 0.05 vs ASCn).

Ingenuity Pathway Analysis (IPA) data identified the most relevant modifications in gene expression in ASCmo. These genes were further validated by real-time PCR (Figure 1). Developmental genes were down-regulated in the ASCmo compare to ASCn since a significantly reduced expression was observed in HOXC10 (Homeobox C10; -1.42 fold), TBX15 (T-box 15; -2.04 fold), ACTA2 (α-smooth muscle actin; -1.42 fold) and SDF-1 (Stromal cell-derived factor 1/CXCL12; -2.32 fold) (Figure 1A).

Moreover, ASCmo presented a significant up-regulation in genes that induce adipocyte lineage commitment or regulates adipogenesis, such as TCF21 (Transcription factor 21; 2.62 fold), PITX2 (Paired-like homeodomain transcription factor 2; 1.88 fold), LIF (Leukemia inhibitory factor; 4.4 fold), TNFRSF11B (Osteoprotegerin; 15.23 fold) and HAPLN1 (Hyaluronan and proteoglycan link protein 1; 2.94 fold). Contrary RUNX2 (Runt-related transcription factor 2; -1.4 fold), that induces osteoblastogenesis, showed a trend towards a decrease expression in ASCmo compared to ASCn (Figure 1B).

There was an up-regulation in the expression of pro-inflammatory molecules such as IL-1β (Interleukin-1β; 4.81 fold), IL-8 (Interleukin-8; 5.9 fold) and CCL2 (monocyte chemotactic protein-1; 3.35 fold), and OLR1 (oxidized low density lipoprotein receptor 1; 7.62 fold) in ASCmo. The dual pro-apoptotic/pro-survival molecule DAPK1 (Death-associated protein kinase 1, -2.40) is down-regulated in ASCmo compared to ASCn (Figure 1C).

### Effects of cardiovascular risk factor clustering

**Figure 2:**
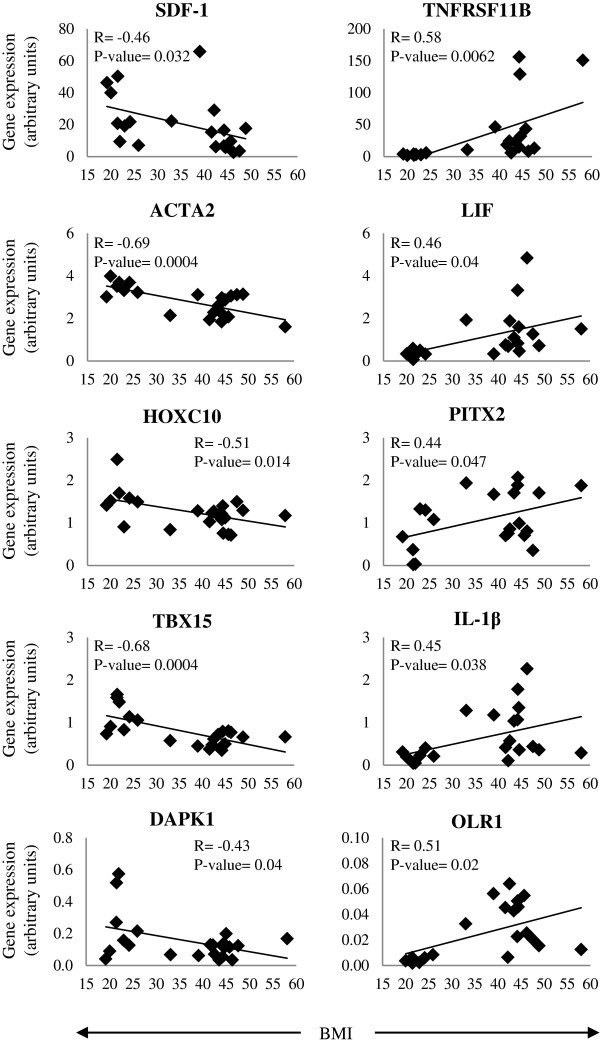
**Correlation between expression of validated genes and body mass index (BMI).** Correlations were determined by linear correlations.

**Figure 3:**
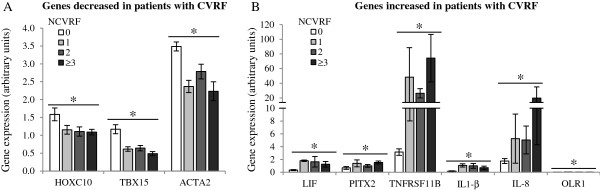
**Cardiovascular risk factors (CVRF) accumulation affects ASC gene expression.** The accumulation of CVRF such as morbid obesity, hypertension, type 2 diabetes, tobacco, previous cardiovascular event and/or dyslipidemia **(A)** decrease and **(B)** increase the expression of various genes in ASC. Correlation significances between gene expression and number of cardiovascular risk factors (NCVRF) were determined as Spearman correlation (* p < 0.05).

**Figure 4:**
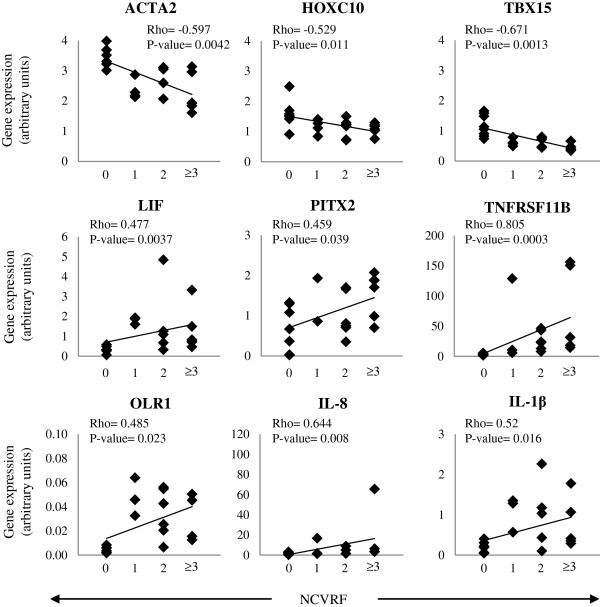
**Correlation between gene expression and the presence of various cardiovascular risk factors (CVRF).** CVRF such as morbid obesity, hypertension, type 2 diabetes, tobacco, previous cardiovascular event and/or dyslipidemia were correlated with gene expression. Correlation significances were determined by Spearman correlation (Rho coefficient). NCVRF, number of cardiovascular risk factors.

Correlation analysis between gene expression and BMI or clustering of cardiovascular risk factors (morbid obesity, high blood pressure, smoking, previous cardiovascular event, dyslipidemia and/or type 2 diabetes) was performed. Expression levels of ACTA2 (R: -0.69), TBX15 (R: -0.68), SDF-1 (R: -0.46) and DAPK1 (R: -0.43) negatively correlated with BMI increase (p < 0.05); whereas LIF (R: 0.46), PITX2 (R: 0.44), TNFRSF11B (R: 0.58), IL-1β (R: 0.45) and OLR1 (R: 0.51) correlated positively (p < 0.05) (Figure 2). Furthermore, ACTA2 (Rho: -5.97), HOXC10 (Rho: -5.29) and TBX15 (Rho: -0.671) negatively correlated with cardiovascular risk factor clustering (p < 0.05); while LIF (Rho: 0–477), PITX2 (Rho: 0.459), OLR1 (Rho: 0.485), TNFRSF11B (Rho: 0.805), IL-1β (Rho: 0.52) and IL-8 (Rho: 0.644) correlated positively (p < 0.05) (Figures 3 and 4).

### SMAD activation differs between ASCn and ASCmo

**Figure 5:**
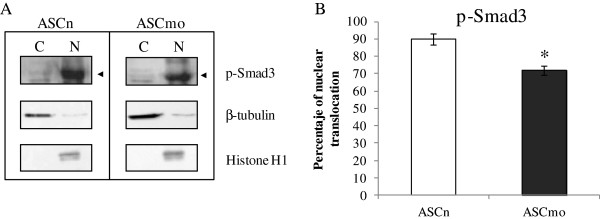
**Nuclear translocation of phospho-Smad3 differs between ASCn and ASCmo. (A)** Western blot analysis of cytoplasmic-membrane (C) and nuclear (N) fraction of ASCn and ASCmo. β-tubulin and Histone H1 were used as a membrane-cytoplasmic and nuclear fraction marker, respectively. **(B)** ASCmo presented a reduced nuclear translocation of phospho-Smad3 compared to ASCn (* p < 0.05 vs ASCn).

Bioinformatic systems biology analysis predicted the SMAD pathway as a potential candidate in the activation/repression of the modified genes in obesity. In order to analyze SMAD pathway activation, we assessed nuclear translocation of phospho-Smad3. We show that ASCmo present a lower activation of SMAD pathway, in fact, phospho-Smad3 nuclear translocation were significantly reduced in the ASCmo (Figure 5).

## Discussion

In this study we demonstrate that obesity affects WAT-resident ASC by differentially regulating their gene expression. The transcription of both genes involved in development as well those involved in adipocyte differentiation is significantly modified. Moreover, ASC from obese patients already show upregulation of inflammatory genes. Interestingly, ASC from obese patients with metabolic syndrome (and clustering of cardiovascular risk factors) show further impairment in their transcriptomic profile than ASC from obese metabolically healthy patients.

ASC in morbidly obese patients show similar ASC surface markers (CD44, CD90, CD29 and CD105) to ASC from non-obese individuals. However, we had previously shown that ASCmo have significantly different cell proliferation, differentiation and angiogenic potential [4]. Here we show that ASC from the subcutaneous WAT of morbidly obese patients adopt a significantly modified expression profile with major changes on genes that regulate cell survival, cell differentiation, tissue integrity and metabolic pathways. Interestingly, the ASC (passage 3; both from non-obese and obese patient abdominal fat) were negative for the surface marker CD34, a marker described for bone marrow cells, hematopoietic stem cells, endothelial progenitor cells and muscle stem cells [13]. In fact, the expression of CD34 by ASC has been a matter of discussion because of contradictory reports and findings [14]. While some authors have described an ASC population that express CD34 [15-17], some others have not been able to detect this marker [1,18,19].

Two highly conserved families of genes involved in embryonic development show down-regulation in ASC from morbidly obese patients: the homeobox (HOXC10) and T-box (TBX15). The HOX code is mainly a “biological fingerprint” of different cell types reflecting a continuation of embryonic patterning [20]. Despite the essential role played by HOX genes in embryonic development, hematopoiesis, cancer, angiogenesis and wound repair, there is limited information on HOX genes in a variety of MSC. Similarly, Tbx15 is one of the fundamental developmental mesodermal transcription factors that is highly expressed in both human and rodent adipose tissue [21]. Here we described for the first time that obesity down-regulates expression of TBX15 and HOXC10 in subcutaneous WAT resident stem cells. The expression of both genes negatively correlates with BMI and cardiovascular risk factor clustering therefore potentially marking a reduced developmental potential of the more differentiated cells of the morbid obesity fat depot. The same result is shown with ACTA2, an ASC gene marker. It has been shown that mice ASC expressing ACTA2 present multilineage differentiation ability compared to ASC that do not express ACTA2 which only display adipogenic commitment [22]. The fact that expression of these three genes are found significantly decreased in ASC from morbidly obese patients indicates that obesity impairs the multipotency of the stem cells homed into their subcutaneous WAT.

Stem cell migration is a key process for development, spontaneous regeneration and homing. It has been described that the CXCR4-SDF-1 axis regulates cellular trafficking [23] and that ASC produce and secrete SDF-1 [24]. Circulating SDF-1 levels are decreased in diet-induced obese mice and in adolescents with elevated waist circumference and BMI measures [25]. In this study we show that ASC in morbidly obese patients show a significant down-regulation in SDF-1 expression, and that the effect is BMI-dependent and correlates with cardiovascular risk factors clustering. These results indicate that obesity seems to impair ASC trafficking and homing.

Given that adipocytes share a common mesenchymal precursor cell with osteoblasts, entrance into the adipocytic or osteoblastic lineage is thought to be mutually exclusive [26]. In fact, our results revealed that the adipogenic lineage is stimulated in ASCmo while osteoblastogenesis is inhibited. We have observed that TCF21 expression, a white WAT-selective marker, is increased in ASCmo. Opposite, RUNX2, an osteoblast marker, presented a non-significant trend to decrease in ASCmo. TCF21 is the first useful positive selection marker for white preadipocytes and plays an important role in adipogenesis. It has been shown that TCF21 suppresses myogenesis [27]. Of interest, PITX2 that is known to upregulate the expression of TCF21 [28], is up-regulated in ASCmo explaining the increase in TCF21 expression.

On the other hand, we detected that ASCmo presented an increased expression in LIF and TNFRSF11B genes. Various studies of LIF in adipose tissue have suggested that adipogenesis could be modulated by this gp130 cytokine at certain stages of development while regulating the expression of genes associated with lipid metabolism [29]. Furthermore, TNFRSF11B (Osteoprotegerin) is a glycoprotein of the tumor necrosis factor (TNF) receptor superfamily that was originally discovered as bone resorption inhibitor [30]. Its expression is high in human WAT, and is increased during 3T3-L1 adipocyte differentiation and under TNF-α and IL-1β stimulation [31]. In humans, plasma TNFRSF11B levels are elevated in patients with cardiovascular risk factors such as type 2 diabetes and abdominal obesity [32]. We have also detected that both LIF and TNFRSF11B levels positively correlate with BMI and the clustering of cardiovascular risk factors. Moreover, RUNX2 is involved in inhibiting adipocyte differentiation and appears to be down-regulated during 3T3-L1 adipocyte differentiation [33] and obesity reduces bone RUNX2 expression leading to bone marrow adiposity [34]. Interestingly, it has been published that ASC from WAT of obese patients show abnormal upregulation of peroxisome proliferator-activated receptor-γ (PPARG), a gene that reveals a high adipogenic potential [12,35]. All together these observations indicate that morbid obesity not only affects ASC multilineage potential but commit ASC to adipogenic lineage.

Recent studies demonstrate that during adipogenesis and development of obesity, the extracellular matrix (ECM) undergoes structural remodeling affecting adipose tissue expandability and functional integrity [36]. Analysis of the secretome of human ASC identified various components of the ECM synthesis and remodeling [37]. The glycosaminoglycan hyaluronan is an integral component of ECM with an important role in organizing and maintaining the integrity of the ECM. Although hyaluronan regulates differentiation of mesenchymal lineage cells to chondrocytes and osteoblasts [38], it has been also shown that hyaluronan increases adipogenesis efficiency likely because it provides an appropriate ECM [39]. In fact, it has been demonstrated that hyaluronan synthesis, in 3T3-L1, appears at the stages where early adipocyte markers develop and correlates with triglyceride accumulation [40]. Interestingly we have detected in ASCmo higher levels of HAPLN1, a member of the hyaluronan and proteoglycan binding link protein gene family that stabilizes the interaction between hyaluronan and versican, two ECM components that contribute to the viscoelastic properties observed during 3T3-L1 adipogenic differentiation [40]. More experiments are needed to address how obesity and cardiovascular risk factors modify the adipose tissue ECM and its expandability.

Obesity is associated with a low-grade chronic inflammation [41]. Paracrine and autocrine signaling in WAT-cells may further enhance inflammation, ultimately increasing proinflammatory cytokines production [41]. Yet, it seems that adipocytes and mainly stromal vascular cells appear to be involved in the local production of such cytokines [11,42]. Here we show that obesity induces inflammatory gene up-regulation already in the stem cells. IL-1β, IL-8 and CCL2 genes are up-regulated in ASCmo. In fact, while we detected that IL-1β increase is affected by BMI, IL-8 expression is correlated with cardiovascular risk factors clustering. IL-1β and IL-8 are considered the prototypical proinflammatory cytokines involved in stimulating the synthesis and secretion of a variety of other interleukins, and also participates in a variety of cell functions [42]. Furthermore, high circulating levels of IL-8 and CCL2 have been found in obese subjects increasing the risk of coronary artery disease and type 1 or 2 diabetes [43]. In fact, IL-8 released from subcutaneous WAT and omental-ASC displayed a positive correlation with BMI [12,44], whereas CCL2 expression was higher in subcutaneous adipocytes of obese than non-obese Pima Indians [9].

Furthermore, we report for the first time that ASCmo show increased expression of OLR1 (LOX-1), a lectin-like receptor for oxidized-LDL. OLR1 is expressed in highly vascularized tissues, plays critical roles in the development of atherosclerosis and related disorders, and is upregulated in hypertensive, dyslipidemic, and diabetic animals and humans [45,46]. A recent study reported that oxidized-LDL-Olr1 interaction induces the generation of reactive oxygen species and stimulates pro-inflammatory gene expression [47]. Thus, the effects of OLR1 in obesity could be related to a complex state of inflammation and adipocyte death. In this study we identified that although OLR1 expression levels in ASC is low, morbid obesity and clustering of cardiovascular risk factors significantly increases ASC OLR1 expression.

Finally, we have observed a significantly reduced expression of DAPK1 in ASCmo. The death-associated protein kinase-1 (DAPK1) is the prototypic member of a family of death-related kinases. However, DAPKs can also act as a survival factors and block apoptosis in response to certain cytokine signals modulating the balance between pro-apoptotic and pro-survival pathways [48].

**Figure 6:**
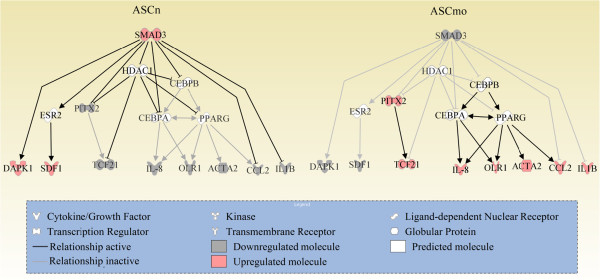
**Hypothetical network proposed to explain the differentially expression pattern between ASCmo and ASCn.** The network was done using Ingenuity Pathways Analysis.

To address the signaling pathways differentially activated in ASCmo, the validated gene expression data was analyzed in silico with the IPA software to obtain the most probable networks and biological pathways. Candidate proteins identified by IPA were validated by western blot. As such, a lower activation of Smad3 was found in the stem cells of the subcutaneous WAT of obese patients. Interestingly, the transcription factor Smad3 plays a key role in the inhibition of adipocyte differentiation and in the development of insulin resistance [49,50]. This integrated bioinformatic systems biology approach led us to propose a network that regulates the differential gene expression pattern in stem cells found in subcutaneous fat of morbidly obese patients (Figure 6).

Our data does not help to explain the obesity paradox. Indeed, studies of patients with chronic heart disease suggest that overweight and obese patients may paradoxically have better outcomes than lean patients. This results may associate adiposity with enhanced cardiovascular autoreparative potential [51]. However our previous results [4] and these here reported indicate that obesity renders ASC less multipotent leaving the paradox unsolved. An alternative not yet fully explored possibility is that obesity could promote MSC recruitment in order to perpetuate adipose tissue formation [2], and consequently lead to increase number of circulating MSC which in turn, upon organ damage, could migrate to the site of injury for repair [52].

## Conclusions

In summary, obesity produces a detrimental effect on its resident stem cells. In fact, the ASC undifferentiated multipotent state is impaired in obese patients with respect to non-obese individuals. This study provides a global systems biology analysis of highly modified genes expressed in ASC from patients with obesity and cardiovascular risk factor clustering that shows how the WAT niche affect resident stem cells. A remaining question that deserves further investigation is whether obesity affects the mesenchymal stem cell transcription potential in the adipose tissue reservoir or stem cells are already conditioned by obesity in the patient’s bone marrow.

## Methods

### Subjects

Subcutaneous abdominal WAT was obtained via surgical resection from morbidly obese patients (BMI > 40 kg/m^2^; n = 16) that underwent bypass gastric surgery, and from non-obese individuals (BMI < 25 kg/m^2^; n = 8) who underwent abdominal lipectomy. Tissues were obtained with informed consent and the study protocol was approved by the Centro Medico Teknon’s Ethical Committee consistent with the principles of the Declaration of Helsinki. Blood samples from all subjects were obtained at the time of intervention in order to evaluate the following biochemical parameters: glucose, triglyceride, total cholesterol, HDL, LDL, urea, total proteins, glutamic oxaloacetic transaminase, glutamic pyruvic transaminase, and creatinine levels. Patients used regular medication as recommended in the guidelines if it was necessary. In order to analyze the effect of cardiovascular risk factors (morbid obesity, high blood pressure, smoking, dyslipidemia, type 2 diabetes and/or previous cardiovascular event), individuals were clustered according to the accumulated cardiovascular risk factors: 0) non-obese individuals, 1) morbid obesity, 2) morbid obesity and one additional risk factor (high blood pressure or dyslipidemia), ≥3) morbid obesity and two or more risk factors (patients with metabolic syndrome).

### ASC isolation and culture

Isolation of ASCmo and ASCn was performed as we have previously described [4]. Briefly, AT was washed, minced and digested with 1 mg/ml Collagenase I-A (Sigma-Aldrich; St. Louis, MO, USA) in Dulbecco’s modified Eagle’s Medium high glucose (Gibco; Life Technologies, Inc., Grand Island, NY, USA) for 1 h at 37°C with gentle agitation. The digested tissue was sequentially filtered through a 100 μm mesh and centrifuged for 10 min at 1200 rpm at room temperature. The supernatant, containing mature adipocytes, was aspirated and the pellet was identified as the stromal vascular fraction (SVF). SVF was resuspended in culture medium (Dulbecco’s modified Eagle’s Medium supplemented with 10% fetal bovine serum, 100 U/ml penicillin and 100 μg/ml streptomycin; Life Technologies, Paisley, UK) and seeded at 2×10^5^cells/cm^2^. Finally, these cells were incubated overnight at 37°C, 5% CO_2_ under hypoxic conditions (1% O_2_); 24 h after incubation, the medium was changed to remove non-adherent cells. These primary adherent cells are referred as ASC passage 0 (cultured for 7 days until they reached 75-90% confluence). For further passages cells were detached by trypsin digestion.

### Flow cytometry characterization

For further characterization, cell surface antigen phenotype was performed on ASCmo and ASCn at passage 0 and passage 3. The following cell-surface epitopes were marked with anti-human antibodies: CD105-fluorescein isothiocyanate (GeneTex), CD90-fluorescein isothiocyanate (FITC), CD29-phycoerythrin (PE), CD44-FITC, CD34-PE-Cy™5 and CD45-PE (BD Biosciences; San Diego, CA, USA). Mouse isotype antibodies served as control (BD Biosciences). Cellular events (30000) were acquired and analyzed by Fluorescence-activated cell sorting using Coulter EPICS XL flow cytometer (Beckman Coulter) running Expo32 software (Beckman Coulter).

### Transcriptomic analysis

The whole genome expression was assessed by independently run microarrays in isolated human ASCmo (n = 3) and ASCn (n = 3) cultured under hypoxic conditions (passage 3) using GeneChip Human Gene 1.0 ST arrays (Affymetrix; Santa Clara, CA, USA). Total cell RNA was extracted using silica-membrane columns (RNesasy Mini Kit, Qiagen; Valencia, CA, USA). RNA quantification was determined spectrophotometrically using the Nanodrop ND-1000 (Thermo Fisher Scientific; Whaltham, MA, USA). RNA quality was measured using Agilent 2100 Bioanalyzer technology (Agilent Technologies; Santa Clara, CA, USA) with the Agilent RNA 6000 Nano Kit (Agilent Technologies). Only RNA samples with RIN > 7 (RNA integrity number) values were chosen for microarray experiments. Using the Ambion WT Expression Kit (Ambion, Life Tecnologies, Paisley, UK) 200 ng of total RNA (mixed with poly-A controls; Affymetrix) were converted to double strand DNA, in two steps, in order to obtain cRNA by an in vitro transcription. Single strand DNA was obtained from 10 ug of cRNA. Then 5.5 ug of single strand DNA were fragmented and labelled with biotin using the WT Terminal Labeling Kit (Affymetrix). Hybridization controls from the Hybridization, Wash and Stain Kit (Affymetrix) were added to the sample. Every sample was hybridized to GeneChip Human Gene 1.0 ST array during 16 h at 45°C and 60 rpm, according to manufacturer’s instruccions. Hybridization, washing, staining and scanning of microarrays were performed according to Affymetrix instructions using the Affymetrix GeneChip 3000 7G System (645 Hybridization Oven, 450 Fluidic Station and GeneChip 3000 7G Scanner).

Raw data were pre-processed with Robust Multiarray Average (RMA) method [53]: background correction, quantile normalization and median polish summarization of the probes. Bioinformatics was used to analyze the extracted log2 expression data. The raw data from the array experiments has been deposited in NCBI’s Gene Expression Omnibus [54] and is accessible through GEO Series accession number (GSE48964; http://www.ncbi.nlm.nih.gov/geo/query/acc.cgi?acc=GSE48964).

Microarray quality control and statistical analyses were performed using the Partek Genomics Suite software (Partek Inc., St Louis, Miossouri, USA). PCA procedure was used to represent samples variability, which defines a group of Principal Components in order to explain the variability existing between the samples. ANOVA, modified by Partek Inc, was used for statistical comparisons between groups. Derived p-values were adjusted for multiple testing with the method of Benjamini and Hochberg [55]. Adjusted p-value, FDR <0.6 and fold change ≥ ±1.5 were used to obtain differential expressed genes. Some of the modified genes were validated using real-time PCR in ASC from additional non-obese individuals (n = 8) and morbidly obese patients (n = 16). Differential expressed genes were associated with biological functions and/or diseases based on literature and the Ingenuity Knowledge Base. Differential expressed genes were analyzed through the use of IPA (Ingenuity® Systems, http://www.ingenuity.com). Right‒tailed Fisher’s exact test was used to calculate a p‒value determining the probability that each biological function and/or disease assigned to that data set is due to chance alone. The data set from our study was used to construct hypothetical protein interaction based on a regularly updated Ingenuity Pathways Knowledge Base. The networks are displayed graphically as nodes (individual proteins) and edges (the biological relationships between the nodes). The relationships include direct and indirect protein interactions.

### Real time PCR validation

In order to validate the generated data, genes of interest that showed a statistically significant difference in expression were validated using custom Taqman Low Density Arrays and real-time PCR assays according to manufacturer’s instructions (Applied Biosystems, Life Technologies). Relative gene expression values were calculated by the ΔΔCt method [56]. The raw gene expression values were normalized according to the expression of TATA-binding protein (TBP) gene.

### Western blotting

ASC subcellular fractionation and western blot analysis were performed to analyze proteins levels and nuclear translocation in non-obese individuals (n = 7) and morbidly obese patients (n = 9).

Subcellular fractionation analysis was performed to evaluate phoshpo-Smad3 nuclear translocation. Nuclear and membrane-cytoplasmic proteins were extracted from ASCn and ASCmo cultured under hypoxic conditions as previously described [57]. The extraction was performed on ice to avoid denaturation of the proteins. Equivalent amounts of proteins were subjected to electrophoresis on 10-12% gels and then transferred to nitrocellulose membrane. After blocking in 5% nonfat milk 1 h at room temperature, blots were incubated with anti-phospho-Smad2/3 goat polyclonal (Santa Cruz Biotechnology; Santa Cruz, CA, USA), anti-β-tubulin rabbit polyclonal (Abcam; Cambridge, UK) and anti-Histone H1 mouse monoclonal (Millipore; Billerica, MA, USA) at 4°C overnight. Secondary antibodies consisted of horseradish peroxidase-conjugated antibodies (Dako; Barcelona, Spain) and were detected using SuperSignal chemiluminescence system (Pierce; Rockford, IL, USA). β-tubulin and Histone H1 were used as a membrane-cytoplasmic and nuclear fraction marker, respectively. Protein expression was determined using Image LabTM Software (BIO RAD). Nuclear translocation was calculated as a relation between nuclear protein vs total protein (nuclear + membrane/cytoplasmic protein) quantification.

### Additional statistical analyses

Results are expressed as means ± standard error mean. Statistical Analyses were performed using StatView® software (SAS Institute Inc.). The statistical significance was assessed by unpaired Student’s *t*-test. Correlation analyses between gene expression and BMI or number of cardiovascular risk factors were determined by linear regression and Spearman correlation, respectively. P-value < 0.05 was considered statistically significant.

## Abbreviations

ASC: Adipose-derived stem cells; ASCn: ASC derived from non-obese individuals; ASCmo: ASC derived from morbid obese patients; BMI: Body mass index; CVRF: Cardiovascular risk factors; FDR: False discovery rate; HDL: High density lipoprotein cholesterol; LDL: Low density lipoprotein cholesterol; MSC: Mesenchymal stem cells; PCA: Principal component analysis; PCR: Polymerase chain reaction; SVF: Stromal vascular fraction; WAT: White adipose tissue.

## Competing interests

The authors declare that they have no competing interest.

## Authors’ contributions

BO performed experiments, interpreted results and wrote the manuscript; GV designed strategy for experimental planning and reviewed the manuscript; SC-L performed experiments and revised the manuscript; JY was in charge of medical patients follow-up; AD-C, CB-L, FM and JH were surgeon in charge of bariatric surgery sample collection; LB planned, evaluated and interpreted experiments and wrote the article. All authors read and approved the final manuscript.

## Supplementary Material

Additional file 1: Table S1 Phenotypical characterization of ASC. Flow cytometry analysis of ASCn and ASCmo at passage 0 and 3 were performed in order to characterize ASC populations. Data are presented as means ± standard error mean. Click here for file

Additional file 2: Figure S1 Principal component analysis (PCA) of genomic expression data from ASC from different donors. Click here for file

Additional file 3: Figure S2 Heat map of genes displaying 1.5-fold difference in expression between ASC individuals groups. Click here for file

## References

[B1] Zuk PA Zhu M Ashjian P De Ugarte DA Huang JI Mizuno H Alfonso ZC Fraser JK Benhaim P Hedrick MH Human adipose tissue is a source of multipotent stem cells Mol Biol Cell 2002 13 4279 4295 10.1091/mbc.E02-02-0105 12475952 PMC138633

[B2] Mansilla E Diaz Aquino V Zambon D Marin GH Martire K Roque G Ichim T Riordan NH Patel A Sturla F Could metabolic syndrome, lipodystrophy, and aging be mesenchymal stem cell exhaustion syndromes? Stem Cells Int 2011 2011 943216 21716667 10.4061/2011/943216 PMC3118295

[B3] Tchkonia T Giorgadze N Pirtskhalava T Thomou T DePonte M Koo A Forse RA Chinnappan D Martin-Ruiz C Von Zglinicki T Kirkland JL Fat depot-specific characteristics are retained in strains derived from single human preadipocytes Diabetes 2006 55 2571 2578 10.2337/db06-0540 16936206

[B4] Oñate B Vilahur G Ferrer-Lorente R Ybarra J Diez-Caballero A Ballesta-Lopez C Moscatiello F Herrero J Badimon L The subcutaneous adipose tissue reservoir of functionally active stem cells is reduced in obese patients FASEB J 2012 26 4327 4336 10.1096/fj.12-207217 22772162

[B5] Gomez-Ambrosi J Catalan V Diez-Caballero A Martinez-Cruz LA Gil MJ Garcia-Foncillas J Cienfuegos JA Salvador J Mato JM Fruhbeck G Gene expression profile of omental adipose tissue in human obesity FASEB J 2004 18 215 217 14630696 10.1096/fj.03-0591fje

[B6] Von Eyben FE Kroustrup JP Larsen JF Celis J Comparison of gene expression in intra-abdominal and subcutaneous fat: a study of men with morbid obesity and nonobese men using microarray and proteomics Ann N Y Acad Sci 2004 1030 508 536 15659836 10.1196/annals.1329.063

[B7] Baranova A Collantes R Gowder SJ Elariny H Schlauch K Younoszai A King S Randhawa M Pusulury S Alsheddi T Obesity-related differential gene expression in the visceral adipose tissue Obes Surg 2005 15 758 765 10.1381/0960892054222876 15978142

[B8] Rodriguez-Acebes S Palacios N Botella-Carretero JI Olea N Crespo L Peromingo R Gomez-Coronado D Lasuncion MA Vazquez C Martinez-Botas J Gene expression profiling of subcutaneous adipose tissue in morbid obesity using a focused microarray: distinct expression of cell-cycle- and differentiation-related genes BMC Med Genomics 2010 3 61 10.1186/1755-8794-3-61 21182758 PMC3022546

[B9] Lee YH Nair S Rousseau E Allison DB Page GP Tataranni PA Bogardus C Permana PA Microarray profiling of isolated abdominal subcutaneous adipocytes from obese vs non-obese Pima Indians: increased expression of inflammation-related genes Diabetologia 2005 48 1776 1783 10.1007/s00125-005-1867-3 16059715 PMC1409820

[B10] Van Tienen FH Van der Kallen CJ Lindsey PJ Wanders RJ Van Greevenbroek MM Smeets HJ Preadipocytes of type 2 diabetes subjects display an intrinsic gene expression profile of decreased differentiation capacity Int J Obes (Lond) 2011 35 1154 1164 10.1038/ijo.2010.275 21326205

[B11] Nair S Lee YH Rousseau E Cam M Tataranni PA Baier LJ Bogardus C Permana PA Increased expression of inflammation-related genes in cultured preadipocytes/stromal vascular cells from obese compared with non-obese Pima Indians Diabetologia 2005 48 1784 1788 10.1007/s00125-005-1868-2 16034612 PMC1409821

[B12] Roldan M Macias-Gonzalez M Garcia R Tinahones FJ Martin M Obesity short-circuits stemness gene network in human adipose multipotent stem cells FASEB J 2011 25 4111 4126 10.1096/fj.10-171439 21846837

[B13] Hombach-Klonisch S Panigrahi S Rashedi I Seifert A Alberti E Pocar P Kurpisz M Schulze-Osthoff K Mackiewicz A Los M Adult stem cells and their trans-differentiation potential–perspectives and therapeutic applications J Mol Med (Berl) 2008 86 1301 1314 10.1007/s00109-008-0383-6 18629466 PMC2954191

[B14] Tarnok A Ulrich H Bocsi J Phenotypes of stem cells from diverse origin Cytometry A 2010 77 6 10 20024907 10.1002/cyto.a.20844

[B15] Festy F Hoareau L Bes-Houtmann S Pequin AM Gonthier MP Munstun A Hoarau JJ Cesari M Roche R Surface protein expression between human adipose tissue-derived stromal cells and mature adipocytes Histochem Cell Biol 2005 124 113 121 10.1007/s00418-005-0014-z 16032396

[B16] Gronthos S Zannettino AC Hay SJ Shi S Graves SE Kortesidis A Simmons PJ Molecular and cellular characterisation of highly purified stromal stem cells derived from human bone marrow J Cell Sci 2003 116 1827 1835 10.1242/jcs.00369 12665563

[B17] Zimmerlin L Donnenberg VS Rubin JP Donnenberg AD Mesenchymal markers on human adipose stem/progenitor cells Cytometry A 2013 83 134 140 23184564 10.1002/cyto.a.22227 PMC4157311

[B18] Musumeci G Lo Furno D Loreto C Giuffrida R Caggia S Leonardi R Cardile V Mesenchymal stem cells from adipose tissue which have been differentiated into chondrocytes in three-dimensional culture express lubricin Exp Biol Med (Maywood) 2011 236 1333 1341 10.1258/ebm.2011.011183 22036733

[B19] De Ugarte DA Morizono K Elbarbary A Alfonso Z Zuk PA Zhu M Dragoo JL Ashjian P Thomas B Benhaim P Comparison of multi-lineage cells from human adipose tissue and bone marrow Cells Tissues Organs 2003 174 101 109 10.1159/000071150 12835573

[B20] Morgan R Hox genes: a continuation of embryonic patterning? Trends Genet 2006 22 67 69 10.1016/j.tig.2005.11.004 16325300

[B21] Gesta S Bluher M Yamamoto Y Norris AW Berndt J Kralisch S Boucher J Lewis C Kahn CR Evidence for a role of developmental genes in the origin of obesity and body fat distribution Proc Natl Acad Sci USA 2006 103 6676 6681 10.1073/pnas.0601752103 16617105 PMC1458940

[B22] Cai X Lin Y Hauschka PV Grottkau BE Adipose stem cells originate from perivascular cells Biol Cell 2011 103 435 447 10.1042/BC20110033 21679159

[B23] Nervi B Link DC DiPersio JF Cytokines and hematopoietic stem cell mobilization J Cell Biochem 2006 99 690 705 10.1002/jcb.21043 16888804

[B24] Kondo K Shintani S Shibata R Murakami H Murakami R Imaizumi M Kitagawa Y Murohara T Implantation of adipose-derived regenerative cells enhances ischemia-induced angiogenesis Arterioscler Thromb Vasc Biol 2009 29 61 66 10.1161/ATVBAHA.108.166496 18974384

[B25] Jung C Fischer N Fritzenwanger M Pernow J Brehm BR Figulla HR Association of waist circumference, traditional cardiovascular risk factors, and stromal-derived factor-1 in adolescents Pediatr Diabetes 2009 10 329 335 10.1111/j.1399-5448.2008.00486.x 19076302

[B26] Sordella R Jiang W Chen GC Curto M Settleman J Modulation of Rho GTPase signaling regulates a switch between adipogenesis and myogenesis Cell 2003 113 147 158 10.1016/S0092-8674(03)00271-X 12705864

[B27] Funato N Ohyama K Kuroda T Nakamura M Basic helix-loop-helix transcription factor epicardin/capsulin/Pod-1 suppresses differentiation by negative regulation of transcription J Biol Chem 2003 278 7486 7493 10.1074/jbc.M212248200 12493738

[B28] Shih HP Gross MK Kioussi C Cranial muscle defects of Pitx2 mutants result from specification defects in the first branchial arch Proc Natl Acad Sci USA 2007 104 5907 5912 10.1073/pnas.0701122104 17384148 PMC1851590

[B29] White UA Stephens JM The gp130 receptor cytokine family: regulators of adipocyte development and function Curr Pharm Des 2011 17 340 346 10.2174/138161211795164202 21375496 PMC3119891

[B30] Kwon BS Wang S Udagawa N Haridas V Lee ZH Kim KK Oh KO Greene J Li Y Su J TR1, a new member of the tumor necrosis factor receptor superfamily, induces fibroblast proliferation and inhibits osteoclastogenesis and bone resorption FASEB J 1998 12 845 854 9657524 10.1096/fasebj.12.10.845

[B31] An JJ Han DH Kim DM Kim SH Rhee Y Lee EJ Lim SK Expression and regulation of osteoprotegerin in adipose tissue Yonsei Med J 2007 48 765 772 10.3349/ymj.2007.48.5.765 17963332 PMC2628141

[B32] Browner WS Lui LY Cummings SR Associations of serum osteoprotegerin levels with diabetes, stroke, bone density, fractures, and mortality in elderly women J Clin Endocrinol Metab 2001 86 631 637 10.1210/jc.86.2.631 11158021

[B33] Zhang YY Li X Qian SW Guo L Huang HY He Q Liu Y Ma CG Tang QQ Down-regulation of type I Runx2 mediated by dexamethasone is required for 3T3-L1 adipogenesis Mol Endocrinol 2012 26 798 808 10.1210/me.2011-1287 22422618 PMC5417096

[B34] Halade GV El Jamali A Williams PJ Fajardo RJ Fernandes G Obesity-mediated inflammatory microenvironment stimulates osteoclastogenesis and bone loss in mice Exp Gerontol 2011 46 43 52 10.1016/j.exger.2010.09.014 20923699 PMC2998554

[B35] Baptista LS Da Silva KR Da Pedrosa CS Claudio-da-Silva C Carneiro JR Aniceto M De Mello-Coelho V Takiya CM Rossi MI Borojevic R Adipose tissue of control and ex-obese patients exhibit differences in blood vessel content and resident mesenchymal stem cell population Obes Surg 2009 19 1304 1312 10.1007/s11695-009-9899-2 19562421

[B36] Lee MJ Wu Y Fried SK Adipose tissue remodeling in pathophysiology of obesity Curr Opin Clin Nutr Metab Care 2010 13 371 376 10.1097/MCO.0b013e32833aabef 20531178 PMC3235038

[B37] Chiellini C Cochet O Negroni L Samson M Poggi M Ailhaud G Alessi MC Dani C Amri EZ Characterization of human mesenchymal stem cell secretome at early steps of adipocyte and osteoblast differentiation BMC Mol Biol 2008 9 26 10.1186/1471-2199-9-26 18302751 PMC2279142

[B38] Huang L Cheng YY Koo PL Lee KM Qin L Cheng JC Kumta SM The effect of hyaluronan on osteoblast proliferation and differentiation in rat calvarial-derived cell cultures J Biomed Mater Res A 2003 66 880 884 12926041 10.1002/jbm.a.10535

[B39] Allingham PG Brownlee GR Harper GS Pho M Nilsson SK Brown TJ Gene expression, synthesis and degradation of hyaluronan during differentiation of 3T3-L1 adipocytes Arch Biochem Biophys 2006 452 83 91 10.1016/j.abb.2006.05.010 16824481

[B40] Zizola CF Julianelli V Bertolesi G Yanagishita M Calvo JC Role of versican and hyaluronan in the differentiation of 3T3-L1 cells into preadipocytes and mature adipocytes Matrix Biol 2007 26 419 430 10.1016/j.matbio.2007.04.002 17513099

[B41] Wellen KE Hotamisligil GS Obesity-induced inflammatory changes in adipose tissue J Clin Invest 2003 112 1785 1788 14679172 10.1172/JCI20514 PMC297006

[B42] Fain JN Release of interleukins and other inflammatory cytokines by human adipose tissue is enhanced in obesity and primarily due to the nonfat cells Vitam Horm 2006 74 443 477 17027526 10.1016/S0083-6729(06)74018-3

[B43] Kim CS Park HS Kawada T Kim JH Lim D Hubbard NE Kwon BS Erickson KL Yu R Circulating levels of MCP-1 and IL-8 are elevated in human obese subjects and associated with obesity-related parameters Int J Obes (Lond) 2006 30 1347 1355 10.1038/sj.ijo.0803259 16534530

[B44] Bruun JM Lihn AS Madan AK Pedersen SB Schiott KM Fain JN Richelsen B Higher production of IL-8 in visceral vs. subcutaneous adipose tissue. implication of nonadipose cells in adipose tissue Am J Physiol Endocrinol Metab 2004 286 E8 E13 13129857 10.1152/ajpendo.00269.2003

[B45] Yan M Mehta JL Hu C LOX-1 and obesity Cardiovasc Drugs Ther 2011 25 469 476 10.1007/s10557-011-6335-3 21881850

[B46] Chui PC Guan HP Lehrke M Lazar MA PPARgamma regulates adipocyte cholesterol metabolism via oxidized LDL receptor 1 J Clin Invest 2005 115 2244 2256 10.1172/JCI24130 16007265 PMC1172230

[B47] Dunn S Vohra RS Murphy JE Homer-Vanniasinkam S Walker JH Ponnambalam S The lectin-like oxidized low-density-lipoprotein receptor: a pro-inflammatory factor in vascular disease Biochem J 2008 409 349 355 10.1042/BJ20071196 18092947

[B48] Lin Y Hupp TR Stevens C Death-associated protein kinase (DAPK) and signal transduction: additional roles beyond cell death FEBS J 2010 277 48 57 10.1111/j.1742-4658.2009.07411.x 19878313

[B49] Tsurutani Y Fujimoto M Takemoto M Irisuna H Koshizaka M Onishi S Ishikawa T Mezawa M He P Honjo S The roles of transforming growth factor-beta and Smad3 signaling in adipocyte differentiation and obesity Biochem Biophys Res Commun 2011 407 68 73 10.1016/j.bbrc.2011.02.106 21356196

[B50] Choy L Derynck R Transforming growth factor-beta inhibits adipocyte differentiation by Smad3 interacting with CCAAT/enhancer-binding protein (C/EBP) and repressing C/EBP transactivation function J Biol Chem 2003 278 9609 9619 10.1074/jbc.M212259200 12524424

[B51] Lavie CJ Milani RV Ventura HO Obesity and cardiovascular disease: risk factor, paradox, and impact of weight loss J Am Coll Cardiol 2009 53 1925 1932 10.1016/j.jacc.2008.12.068 19460605

[B52] Bays HE Adiposopathy is “sick fat” a cardiovascular disease? J Am Coll Cardiol 2011 57 2461 2473 10.1016/j.jacc.2011.02.038 21679848

[B53] Irizarry RA Bolstad BM Collin F Cope LM Hobbs B Speed TP Summaries of affymetrix GeneChip probe level data Nucleic Acids Res 2003 31 e15 10.1093/nar/gng015 12582260 PMC150247

[B54] Edgar R Domrachev M Lash AE Gene expression omnibus: NCBI gene expression and hybridization array data repository Nucleic Acids Res 2002 30 207 210 10.1093/nar/30.1.207 11752295 PMC99122

[B55] Benjamini Y Hochberg Y Controlling the false discovery rate: a practical and powerful approach to multiple testing J R Stat Soc Ser B Methodol 1995 57 289 300

[B56] Livak KJ Schmittgen TD Analysis of relative gene expression data using real-time quantitative PCR and the 2(−delta delta C(T)) method Methods 2001 25 402 408 10.1006/meth.2001.1262 11846609

[B57] Arderiu G Pena E Aledo R Juan-Babot O Badimon L Tissue factor regulates microvessel formation and stabilization by induction of chemokine (C-C motif) ligand 2 expression Arterioscler Thromb Vasc Biol 2011 31 2607 2615 10.1161/ATVBAHA.111.233536 21868706

