# Supplementary figures and images for: Stem cells isolated from adipose tissue of obese patients show changes in their transcriptomic profile that indicate loss in stemcellness and increased commitment to an adipocyte-like phenotype
Source: BMC Genomics. 2013 Sep 16;14:625. doi: 10.1186/1471-2164-14-625 (PMC3848661; doi:10.1186/1471-2164-14-625)

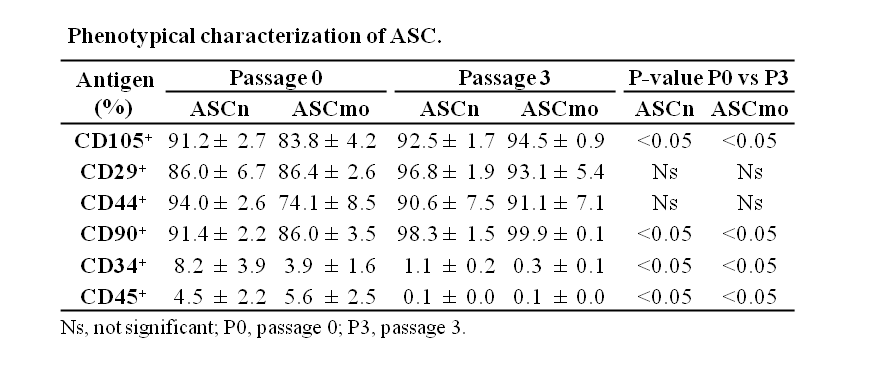

Supplement: Additional file 1: Table S1 — Phenotypical characterization of ASC. Flow cytometry analysis of ASCn and ASCmo at passage 0 and 3 were performed in order to characterize ASC populations. Data are presented as means ± standard error mean. [file 1471-2164-14-625-S1.tiff]

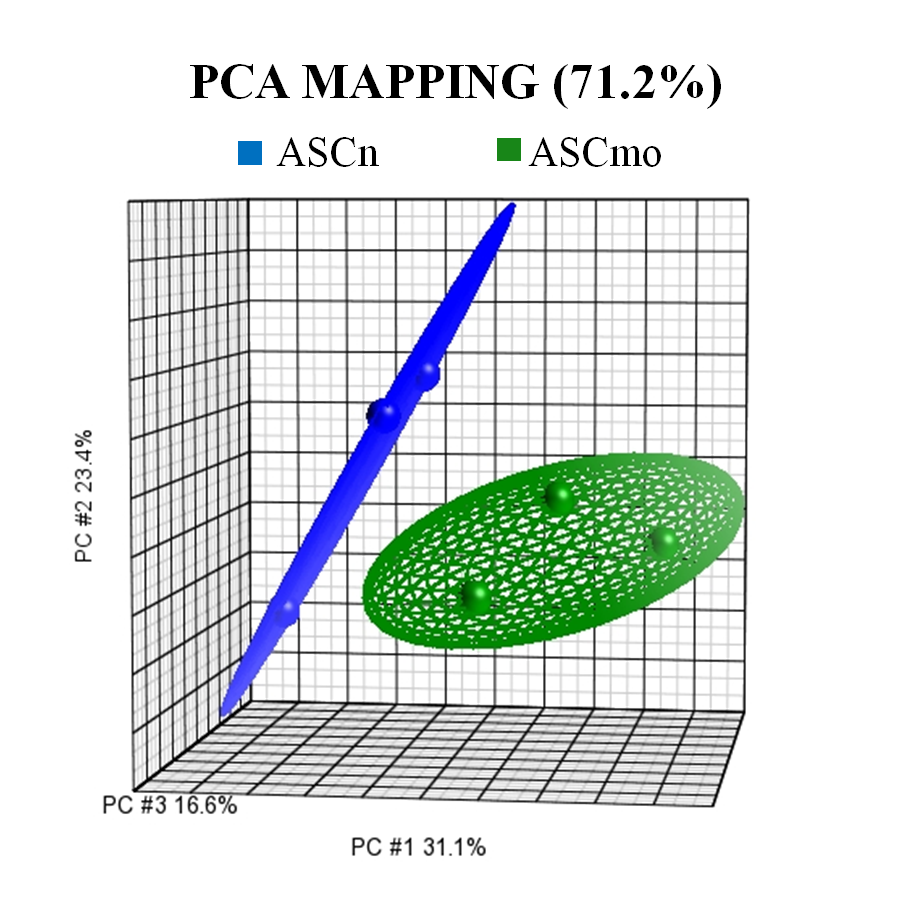

Supplement: Additional file 2: Figure S1 — Principal component analysis (PCA) of genomic expression data from ASC from different donors. [file 1471-2164-14-625-S2.tiff]

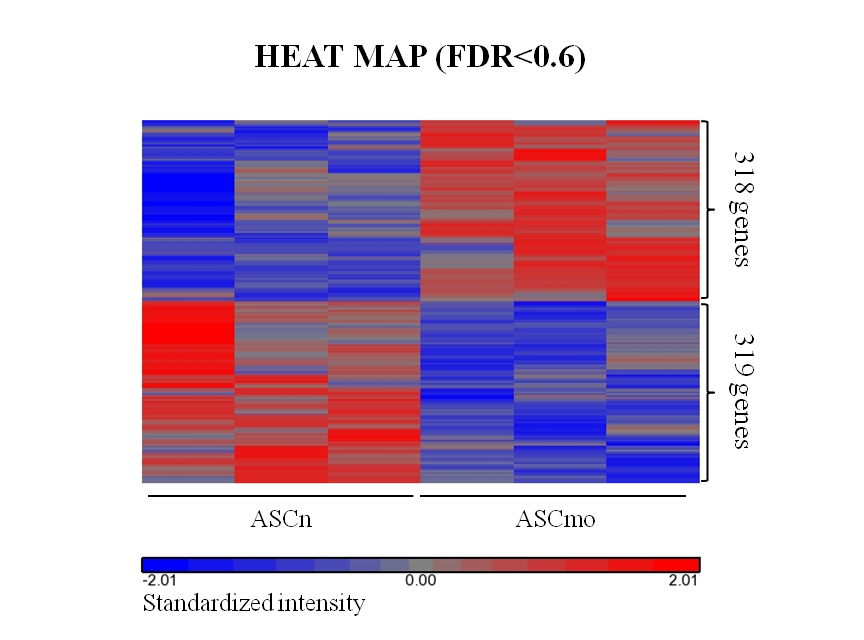

Supplement: Additional file 3: Figure S2 — Heat map of genes displaying 1.5-fold difference in expression between ASC individuals groups. [file 1471-2164-14-625-S3.tiff]
